# Exploring the nexus between MYH9 and tumors: novel insights and new therapeutic opportunities

**DOI:** 10.3389/fcell.2024.1421763

**Published:** 2024-08-01

**Authors:** Zixuan Gou, Difei Zhang, Hongliang Cao, Yao Li, Yunkuo Li, Zijian Zhao, Ye Wang, Yishu Wang, Honglan Zhou

**Affiliations:** ^1^ Key Laboratory of Pathobiology, Ministry of Education, Jilin University, Changchun, China; ^2^ Department of Urology II, The First Hospital of Jilin University, Changchun, China

**Keywords:** MYH9, NM IIA, tumor, clinical translations, therapeutic target

## Abstract

The myosin heavy chain 9 (MYH9) gene, located on human chromosome 22, encodes non-muscle myosin heavy chain IIA (NM IIA). This protein is essential to various cellular events, such as generating intracellular chemomechanical force and facilitating the movement of the actin cytoskeleton. Mutations associated with thrombocytopenia in autosomal dominant diseases first highlighted the significance of the MYH9 gene. In recent years, numerous studies have demonstrated the pivotal roles of MYH9 in various cancers. However, its effects on cancer are intricate and not fully comprehended. Furthermore, the elevated expression of MYH9 in certain malignancies suggests its potential as a target for tumor therapy. Nonetheless, there is a paucity of literature summarizing MYH9’s role in tumors and the therapeutic strategies centered on it, necessitating a systematic analysis. This paper comprehensively reviews and analyzes the pertinent literature in this domain, elucidating the fundamental structural characteristics, biological functions, and the nexus between MYH9 and tumors. The mechanisms through which MYH9 contributes to tumor development and its multifaceted roles in the tumorigenic process are also explored. Additionally, we discuss the relationship between MYH9-related diseases (MYH9-RD) and tumors and also summarize tumor therapeutic approaches targeting MYH9. The potential clinical applications of studying the MYH9 gene include improving early diagnosis, clinical staging, and prognosis of tumors. This paper is anticipated to provide novel insights for tumor therapy.

## Graphical Abstract

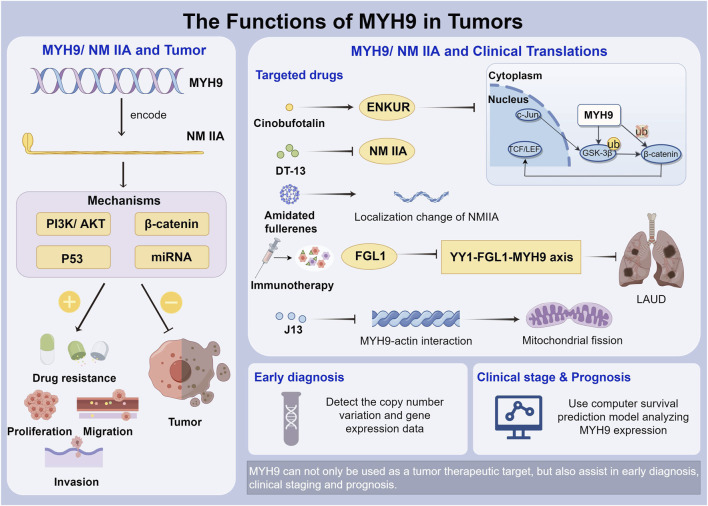

## 1 Introduction

The extensive protein superfamily of myosins plays a vital role in converting the energy released during ATP hydrolysis into conformational changes that drive molecular motion. Specifically, class II myosins assemble into filaments, creating force and tension through the binding of their motor structural domains to actin filaments (Asensio-Juarez et al., 2020). Class II myosins can be broadly classified into muscle types, including skeletal and cardiac muscle, as well as smooth muscle, and non-muscle myosins (Cao et al., 2022). Non-muscle class II myosins can be divided into three variants based on their heavy chains. The MYH9 gene encodes the non-muscle myosin heavy chain A isoform, known as NM IIA which is a member of the myosin family, binds to actin, utilizes magnesium-dependent ATP hydrolysis to generate mechanical force, and is often referred to as an actin molecular motor (Pecci et al., 2018).

The MYH9 gene is increasingly associated with cancer, as evidenced by multiple recent studies (Garlapati et al., 2024). It assumes varying roles in different tumors, serving as either a tumor-promoting or a tumor-suppressing gene (Islam et al., 2023). Over 90% of deaths in patients with malignant tumors are linked to tumor metastasis (Bakir et al., 2020). Tumor cell migration and infiltration are critical components of the metastatic process (Liu G. et al., 2021). Alterations in tumor cell adhesion trigger metastasis from the primary site to distant organs, and cells subsequently move from one metastatic site to another (Manfioletti and Fedele, 2022). Intracellular structural proteins play a vital role in cell migration by facilitating dynamic cytoskeletal assembly and energy provision (Fletcher and Mullins, 2010), ultimately resulting in a poor prognosis (Bera et al., 2022). Moreover, the MYH9 gene contributes to the development of drug resistance in tumors (Ouyang et al., 2022), and although some strategies exist to address this issue, additional research is required to assess their universality and identify potentially superior alternatives. Additionally, the mechanism underlying drug resistance remains a focus of research.

Therapeutic interventions effective against MYH9 have been developed, which encompass Cinobufotalin (CB) (Liu et al., 2022), ENKUR (Hou et al., 2022a), saponin monomer 13 (DT-13) (Du H. et al., 2016), and immunotherapy. Moreover, miRNAs (Hart et al., 2019) and aminated fullerene (Huo et al., 2022) have also exhibited certain effects and potential in specific malignancies.

This review presents an overview of MYH9’s role in tumors and introduces potential therapeutic targets, aiming to offer novel insights for exploring tumor mechanisms and treatment.

## 2 Structural features of MYH9 gene and protein

The MYH9 gene is situated on human chromosome 22 q12-13, comprising 41 exons with an approximate length of 107 kbp (Wang et al., 2023). The first exon is non-translated, and the open reading frame spans from exon 2 to exon 41, encoding a 1,960 amino acid protein called non-muscle myosin heavy chain IIA (NMMHC IIA). This protein is a broadly expressed cytoplasmic myosin involved in numerous processes that necessitate intracellular chemomechanical force generation and actin cytoskeletal translocation (Olson, 2022; Safiullina et al., 2022). Its function is regulated through the phosphorylation of its 20 kDa light and heavy chains and interactions with other proteins. Its structure includes a consistent segment with a molecular weight of 226.59 kD (Asensio-Juarez et al., 2020; Allen et al., 2022; An et al., 2022). One of the key catalysts for NMMHC IIA assembly, Rho-kinase 1 (ROCK1), is a downstream effector of RhoA that can phosphorylate the light chain (RLC) to control its activity and promote the unfolding of NMMHC IIA into an assembly-competent form. This NMMHC IIA, which is assembly-competent, dimerizes to produce NM IIA (Tolue Ghasaban et al., 2023a; Tolue Ghasaban et al., 2023b; Dai et al., 2023; van der Krogt et al., 2023).

The main structural element of the actin cytoskeleton is non-muscle myosin II (NM II). Three separate genes (MYH9, MYH10, and MYH14) encode three different types of NMHCs (IIA, IIB, and IIC), which together make up the NM II isoforms known as NM IIA, NM IIB, and NM IIC (Bourdais et al., 2023; Rouaud et al., 2023). NM IIA is a hexameric molecule composed of a heavy chain dimer (230 kDa), two regulatory light chains (20 kDa) that modulate myosin activity, and two essential light chains (17 kDa) that reinforce the heavy chain structure. Each heavy chain embodies the typical structure of class II myosin, comprising two distinct structural domains: the N-terminal head structural domain and the C-terminal tail structural domain (Parker et al., 2014; Gao et al., 2022). The motor structural domain is situated at the N-terminus, housing the actin-binding site and the ATP-hydrolyzing structural domain. This domain is encoded by exons 2–19. Exon 20 encodes the neck, a region where light chains bind, facilitating the conversion of force generated by the motor domain into movement through rotation. Exons 21 to 40 encode the coiled coil of NM IIA, a region responsible for facilitating the dimerization of the primary encoded product NMHC IIA, to form the NM IIA hexamer. Exon 41 encodes the final 34 amino acids of the non-helical tail, and this region is highly distinct between isoforms. It plays a key role in regulating filament formation through protein interactions and/or phosphorylation (Barvitenko et al., 2021; Carmena, 2021; Cowan et al., 2022). Ser, Thr, and Tyr residues are phosphorylated or dephosphorylated by RLC, which primarily controls the activation and inactivation of NM IIA (Brito et al., 2023). Through antiparallel interactions in their tail regions, NM IIA molecules group together to form NM IIA bipolar filaments that are around 300 nm in length (Saito et al., 2021). The structural domains of the NM IIA motor are free to interact with polymerized actin because they are pointed outward from the polymer. Stress fibers or more dynamically cross-linked actin networks are created when NM IIA polymers bond to actin filaments (Weissenbruch et al., 2022; Brito et al., 2023; Das et al., 2023). NM IIA can be released because ATP binding detaches it from actin. ATP is hydrolyzed by myosin motor heads and reattached to actin which filament contraction is aided by the release of phosphoric acid (Pi), that causes a conformational shift (Garrido-Casado et al., 2021; Halder et al., 2021).

There is mounting evidence that NM II family members, notably NM IIA, are key players in the development of cancer through bivalent binding and actin filament attachment (Weissenbruch et al., 2021; Peng et al., 2022; Weissenbruch et al., 2022).

## 3 Biological functions of MYH9

The MYH9 gene encodes the NM IIA, a broadly expressed cytoplasmic myosin that serves a significant biological role in the human body (Yamamoto et al., 2021; Bai et al., 2022; Ren et al., 2022).

### 3.1 Cell movement and cell mechanics

Cell motility and mechanics reflect how cells respond to external stimuli by migrating, contracting, and deforming. In these processes, MYH9 serves the following functions:

CELL CONTRACTION AND DEFORMATION: MYH9 participates in cell contraction and deformation by interacting with actin in the cytoskeleton (Lin et al., 2017). It modulates the organized assembly and disassembly of actin to regulate cell contraction and deformation (Heuze et al., 2019; Rouaud et al., 2023; Tsukita et al., 2023). MYH9 activity influences the generation and control of intracellular mechanical tension, thereby impacting cell morphology and movement (Barnea et al., 2016; Law et al., 2023).

FIBRONECTIN FORMATION: MYH9 is additionally engaged in the formation and depolymerization of fibronectin. Fibronectin is a critical cytoskeletal component that influences the regulation of cellular motility and mechanical characteristics. MYH9 interacts with fibronectin and enhances its polymerization or depolymerization, thus governing the dynamic reconfiguration of the cytoskeleton and alterations in cell morphology (Breckenridge et al., 2009; Garrido-Casado et al., 2021).

CELL ADHESION AND MOVEMENT: MYH9 participates in cell adhesion and movement processes. Cells adhere to the extracellular matrix or other cell surfaces, subsequently utilizing the mechanical forces of actin and MYH9 to facilitate processes like protrusion, cellular movement, and object phagocytosis (Ivanov et al., 2009; Wang Q. et al., 2022).

MYH9 plays a crucial role in cell motility and mechanics by participating in cell contraction, deformation, fibronectin formation, adhesion, and movement processes (Liu et al., 2017; Zhou et al., 2020a). Consequently, it impacts cell morphology, mechanical properties, and influences cell function and behavior.

### 3.2 Relationship between MYH9 and platelets

A close connection exists between MYH9 and platelets. Platelets, small cell fragments in the blood, primarily participate in hemostasis and thrombosis. MYH9 exerts a significant influence on platelets, contributing to the following aspects:

PLATELET CONTRACTION: MYH9 participates in the regulation of platelet contraction, a critical physiological process enabling platelets to aggregate and form thrombi in response to vascular injury. This process is essential for hemostasis. MYH9 interacts with actin in platelet cells, promoting platelet contraction and enhancing thrombotic force by controlling the organized assembly and disassembly of actin (Pal et al., 2020; Rogerson et al., 2021).

PLATELET MORPHOLOGY: MYH9 is essential for platelet structure and shape. By participating in the regulation of the actin backbone, MYH9 influences platelet morphology and stability. Mutations and abnormal expression of MYH9 can result in changes in platelet structures and abnormalities, ultimately affecting its functions and causing dysfunction (Baumann et al., 2022; Cao et al., 2022).

MYH9 plays a role in platelet contraction, modulation of platelet shape, and is associated with thrombocytopenia, among other crucial platelet functions which could have significant implications for platelet function, as well as the development and progression of thrombotic diseases.

## 4 Relationship between MYH9 and neoplastic diseases

**FIGURE 1:**
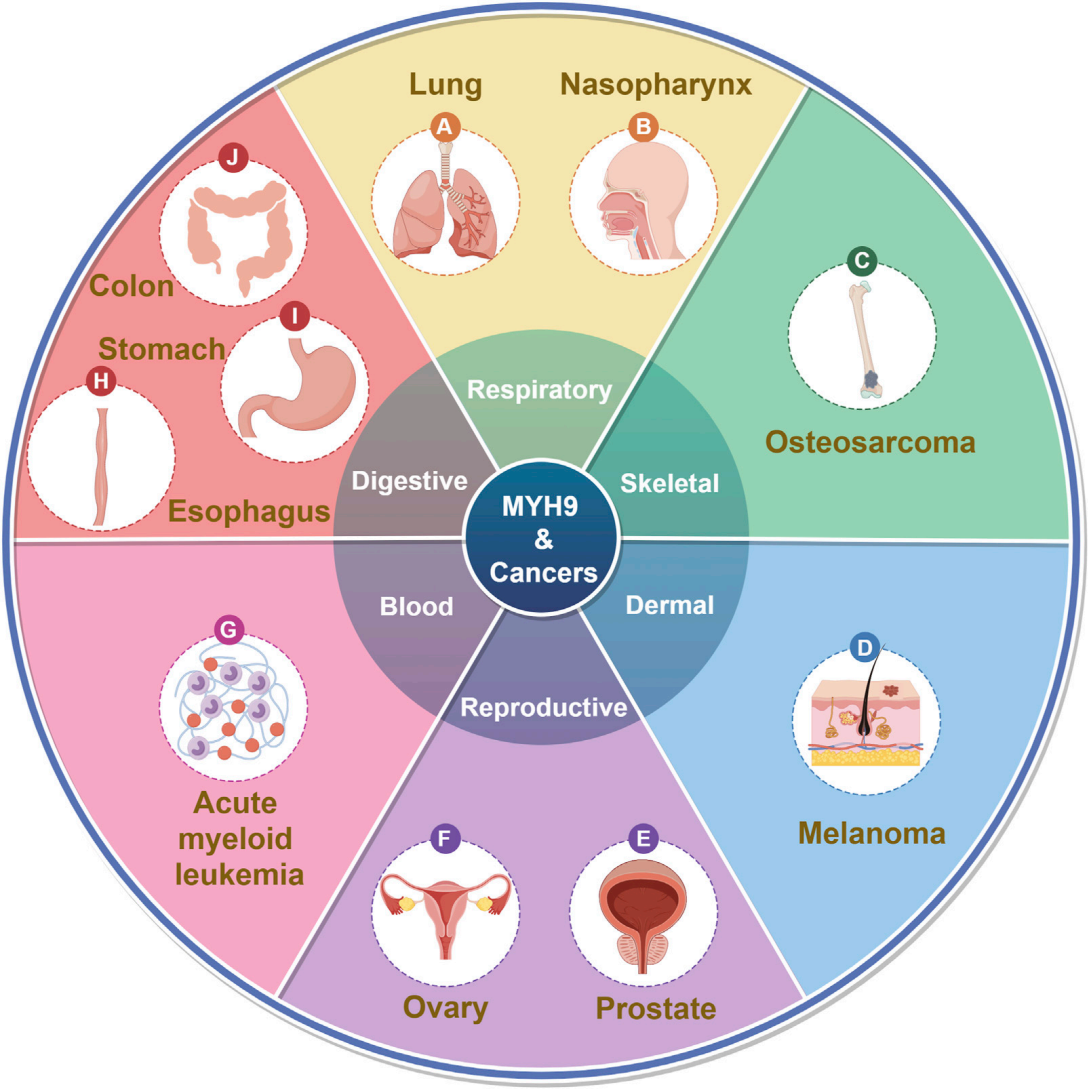
MYH9 and cancers. (By Figdraw).

MYH9 was initially identified due to abnormalities associated with MYH9 mutations, including conditions like May-Hegglin anomaly (MHA) (Sung et al., 2014), Epstein syndrome (EPS) (Pal et al., 2020), Fechtner syndrome (FTNS) (Li X. et al., 2023), Sebastian syndrome (SBS) (Shin et al., 2011) and other autosomal dominant disorders leading to thrombocytopenia (Althaus and Greinacher, 2009; Furlano et al., 2019). These conditions collectively fall under the term MYH9-related disorders or MYH9-RD. In recent years, an increasing number of studies have shown that MYH9 plays a significant role in cancer (Figure 1; Table 1).

**TABLE 1:** Summary of MYH9’s functions in the malignant characteristics of tumors.

Malignant characteristics	Tumor types	Expression	Mechanisms	References
Growth and metastasis	Hepatocellular carcinoma	Upregulated	Activate PI3K/AKT signaling pathway	Zhao et al. (2022a)
Cell proliferation and differentiation	Acute myeloid leukemia	Upregulated		Cui et al. (2022)
Tumorigenesis and low survival rates	Esophageal cancer	Upregulated		Kai et al. (2022)
Sunitinib resistance	Clear cell renal cell carcinoma	Upregulated		Xu et al. (2022)
Malignant progression and resistance to chemotherapy	Nasopharyngeal carcinoma	Upregulated	HMGA1 induces MYH9-dependent ubiquitylation of GSK-3β through the PI3K/Akt/c-Jun signaling pathway	Liu et al. (2019b)
The postoperative recurrence	Esophageal squamous cell carcinoma	Upregulated	Activate GSK 3β/β-catenin signaling pathway	Li et al. (2023b)
Cell migration and invasion	Gastric cancer	Upregulated	Induces deubiquitination of β-catenin through the process of EMT	Liu et al. (2020a)
EMT	Prostate cancer	Upregulated	Mediate ubiquitination and degradation of GSK 3β	Gao et al. (2022)
	Nasopharyngeal carcinoma	Upregulated	Reduce the recruitment of the E3 ligase UBE3A and hinder the UBE3A-mediated degradation of p53 through ubiquitination	Hou et al. (2022b)
EMT and cisplatin resistance	Lung adenocarcinoma	Upregulated	Recruit the deubiquitinating enzyme USP7	Liu et al. (2022)
Cell cycle and EMT signals	Lung and colorectal cancers	Upregulated	Recruit deubiquitination enzyme USP7, inhibiting the degradation of the c-Myc	Hou et al. (2022a)
Proliferation and metastasis	Triple-negative breast cancer	Upregulated	EIF6-224aa interacts with MYH9 and decreases MYH9 degradation by inhibiting the ubiquitin-proteasome pathway and subsequently activating the Wnt/beta-catenin pathway	Li et al. (2022a)
Cell viability and invasive ability	Osteosarcoma	Upregulated	MRPL23-AS1 correlates with MYH9, while conversely correlated with miR-30b, suggesting that the regulatory axis of MRPL23-AS1/miR-30b/MYH9 does exist	Zhang et al. (2021b)
Proliferation and carcinogenesis	Colorectal cancer	Upregulated	Destabilize p53 pre-mRNA by recruiting hnRNPA2B1 in the nucleus	Liu et al. (2021b)
Inflammatory response	Gastric cancer	Upregulated	MYH9-p53-RhoA regulatory feedback loop	Yang et al. (2019)
	Colorectal cancer	Upregulated	circ_0000395 improve the production of MYH9 by chelating miR-432-5p	Fan et al. (2023)
Cell growth and metastasis	Pancreatic ductal adenocarcinoma	Upregulated	circSTX6 controls MYH9 expression by circSTX6/miR-449b-5p and circSTX6/CUL2/HIF1A signaling pathway. MYH9 can interact with CUL2	Gautam et al. (2023), Meng et al. (2022)
Cisplatin resistance and immune response	Non-small cell lung cancer	Upregulated	miR-138-5p/MYH9 axis	Xu et al. (2021), Wang et al. (2022b)
Differentiation and type resistance	Thyroid carcinoma	Upregulated	miR-370-3p/MYH9 axis	Chen et al. (2021)
Glycolysis	Gastric cancer	Upregulated	circ-NRIP1 increases MYH9 expression *via* miR-186-5p	Liu et al. (2020b)
Glycolysis, cell migration, and invasion	Gastric cancer	Upregulated	miR-204-5p/MYH9 axis	Fang et al. (2020)
Cancer growth	Gastric cancer	Upregulated	miR-9-5p/MYH9 axis	Liu et al. (2020c)
Cell proliferation and apoptosis	Non-small cell lung cancer	Upregulated		Alanazi et al. (2023)
Proliferation	Lung cancer	Upregulated	YY1-FGL1-MYH9 axis	Tang et al. (2022)
Migration, invasion, deformation, and proliferation	Lung cancer	Upregulated	MICAL2, a tumor promoter, as a nucleoplasmic shuttle protein dependent on MYH9 and its C-terminal fragment	Ivanov et al. (2009)
Proliferation, migration, invasion, metastasis, and cisplatin resistance	Ovarian cancer	Upregulated	Bind to the MYH10 protein, recruiting deubiquitin-specific protease 45	Liu et al. (2023a)
Cell migration	Esophageal squamous cell carcinoma	Upregulated	GSK3β/β-catenin signaling	Li et al. (2023b)
	Prostate cancer	Upregulated	Act as a novel androgen receptor co-repressor	Liu et al. (2021c)
Cell division, adhesion, and migration	Acute myeloid leukemia	Upregulated	Enhanced actinomyosin contractility	Chang et al. (2020)
Metastasis	Colorectal cancer	Upregulated	Interact with ATG9B	Zhong et al. (2021)
DNA synthesis		Upregulated	dNTPs augment the thermal stability of MYH9, then propel cells into the S phase	Nangia-Makker et al. (2022)
Cell activity	Colon cancer	Upregulated		Lee et al. (2023a)
Cell proliferation and migration	Cervical squamous cell carcinoma	Upregulated	Regulate the content of lipid droplets by binding to ARP2/3	Zhao et al. (2022b)
Temozolomide resistance, cell growth, invasion and migration	Glioma	Upregulated	Interact with GSK-3β, leading to the inhibition of GSK-3β protein expression through ubiquitination	Chen et al. (2023) Que et al. (2021)
Tumorigenesis	HER2+ breast cancers	Downregulated		Alanazi et al. (2023)
Migration, invasion, tumor growth and metastasis	Melanoma	Downregulated	Influence EMT, the ERK signaling pathways and the tumor microenvironment by modulating leukocyte and macrophage infiltration	Singh et al. (2020)
Invasion	Head and neck squamous cell carcinoma	Downregulated	Increase survival with low-risk mutp53	Coaxum et al. (2017)
	Ovarian clear cell carcinoma	Downregulated	Interaction of membrane ebp 50	Nakagawa et al. (2023)
Resistance to levatinib	Hepatocellular carcinoma	Upregulated	NOTCH pathway	Yang et al. (2023)
Stromal stiffness-mediated Metformin resistance	Hepatocellular carcinoma	Upregulated	Increase extracellular matrix stiffness	Gao et al. (2023)
Docetaxel resistance	Prostate cancer	Upregulated	A positive feedback loop of lincROR/MYH9/HIF1α	Jiang et al. (2023)
5-FU resistance	Colon cancer	Upregulated	AMPK/mTOR pathway	Wang et al. (2021)
Osimotinib resistance	Lung adenocarcinoma	Upregulated	MYH9-RETA fusion and T790M deletion in plasma ctDNA	Sun et al. (2020)
Cisplatin resistance	Neuroblastoma	Upregulated		Xu et al. (2020), Belhajova et al. (2022), Li et al. (2022b)
Cisplatin resistance	Nasopharyngeal carcinoma	Upregulated	Interact with FOXO1	Li et al. (2019)

**FIGURE 2:**
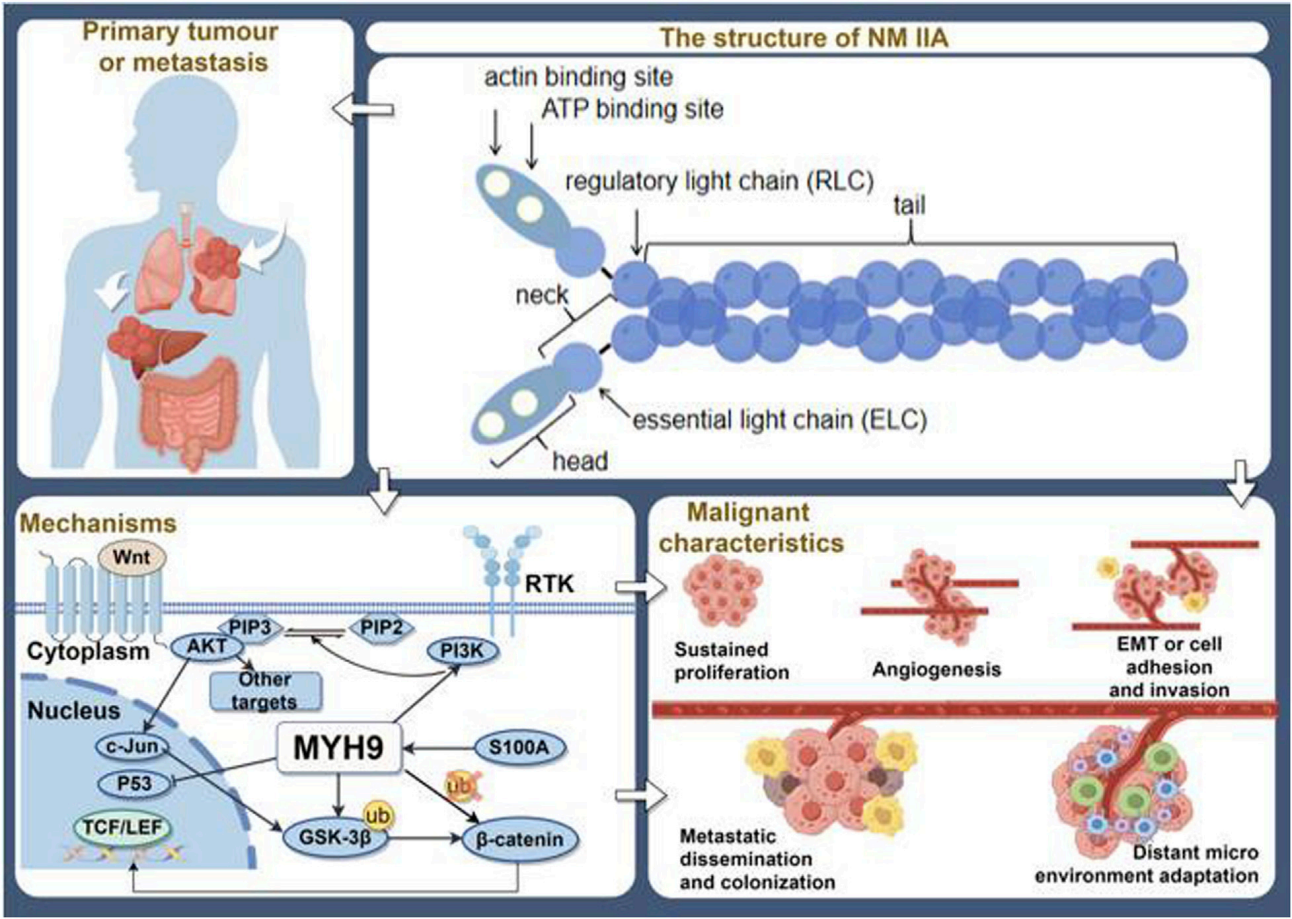
The overview of MYH9′ s roles in tumors. (By Figdraw).

MYH9 can participate in processes like cytoskeletal reorganization and migration as an oncogene. It is associated with clinical staging, histological type, and tumor drug resistance (Yang et al., 2023). It is progressively emerging as a potential molecular marker that offers new insights for tumor prognosis assessment and personalized treatment. Simultaneously, it can also function as a tumor suppressor (Wang et al., 2019). These two contrasting roles are not contradictory but rather depend on the specific type of cancer (Figure 2).

### 4.1 MYH9 is involved in tumor development as an oncogene

Increased expression of the MYH9 gene is frequently observed in respiratory neoplasms, including lung cancer (Xu et al., 2021; Tang et al., 2022), reproductive tumors such as ovarian cancer (Liu L. et al., 2019; Liu L. et al., 2023), prostate cancer (Gao et al., 2022), as well as digestive system tumors like hepatocellular carcinoma (HCC) (Zhang F. et al., 2021; Hou et al., 2022a; Zhao R. et al., 2022), colorectal cancer (CRC) (Song M. et al., 2022) and esophageal cancer (EC) (Kai et al., 2022; Li Q. et al., 2023). Hematologic tumors, such as acute myeloid leukemia (AML) (Cui et al., 2022), and other malignancies also exhibit high MYH9 expression, contributing to tumor progression through diverse mechanisms (Figure 1).

#### 4.1.1 Specific mechanisms by which MYH9 affects tumorigenesis and development

##### 4.1.1.1 PI3K/AKT pathway

MYH9 overexpression can inhibit the PI3K/AKT signaling pathway, leading to increased p-PI3K and p-AKT levels, which in turn participate in tumor cell progression (Xiong et al., 2012; Xiong et al., 2021; Zhang et al., 2023). In a study by Zhao et al., it was found that nucleosome assembly protein 1-like 5 (NAP1L5) inhibits the PI3K/AKT/mTOR signaling pathway in HCC by down-regulating MYH9, leading to therapeutic effects (Zhao R. et al., 2022). Enrichment analysis and a protein-protein interaction network with related genes indicated that Talin1 and MYH9 may bind and interact with each other involved in the process of AML. It can regulate significant signaling pathways in hematological tumors, including PIK3/AKT, promoting tumor cell proliferation and facilitating differentiation (Cui et al., 2022). Another study suggested that MYH9 significantly activates the PI3K/AKT/mTOR axis in esophagus cancer (EC) cells, promoting tumorigenesis. It is upregulated in EC patients with low survival rates (Kai et al., 2022). Clear cell renal cell carcinoma (ccRCC) poses a significant global health threat due to its heterogeneity, which hampers treatment success and results in poor survival outcomes. Mechanistic studies have revealed that MYH9 can fulfill these crucial roles *via* the AKT signaling pathway. Furthermore, the MYH9/AKT axis influences how ccRCC cells respond to Sunitinib treatment and may serve as a biomarker for assessing the benefit of Sunitinib in ccRCC patients (Xu et al., 2022). Que et al. demonstrated that HMGA1 can induce MYH9-dependent ubiquitylation of GSK-3β through the PI3K/Akt/c-Jun signaling pathway, thereby promoting the malignant progression of nasopharyngeal carcinoma and its resistance to chemotherapy (Liu Y. et al., 2019).

##### 4.1.1.2 β-catenin/MYH9 pathway

LincROR is a significant oncogenic long non-coding RNA (Shao et al., 2020). In a study by Jiang et al., it was demonstrated that exosome-mediated lincROR activates a positive feedback loop involving β-catenin and hypoxia-inducible factor 1-alpha (HIF1α) by targeting the MYH9 protein. This activation leads to Docetaxel resistance in prostate cancer (PCa) (Jiang et al., 2023). Moreover, the MYH9-mediated GSK 3β/β-catenin signaling pathway can promote the postoperative recurrence of EC (Li Q. et al., 2023). In gastric cancer (GC), MYH9-induced deubiquitination of β-catenin promotes tumor cell migration and invasion through the process of epithelial-mesenchymal transition (EMT) (Liu J. et al., 2020). In PCa, MYH9-mediated ubiquitination and degradation of GSK 3β can also activate the β-catenin signaling pathway and induce associated epithelial-mesenchymal transition (EMT) too (Gao et al., 2022). Hou et al. discovered that by inhibiting the β-catenin/MYH9 signaling pathway, the recruitment of the E3 ligase UBE3A is reduced. This reduction hinders the UBE3A-mediated degradation of p53 through ubiquitination. Consequently, the EMT signaling pathway is deactivated, preventing nasopharyngeal carcinoma metastasis (Hou et al., 2022b). Furthermore, a comparable mechanism was observed in other types of tumors, including HCC (Hou et al., 2022a), lung adenocarcinoma (LUAD) (Liu et al., 2022), diffuse large B-cell lymphoma (Hu et al., 2022), triple-negative breast cancer (Li Y. et al., 2022), and osteosarcoma (Zhang H. et al., 2021).

##### 4.1.1.3 p53 protein

p53 is a crucial tumor suppressor known for its role in reducing EMT (Hou et al., 2022b). Studies have demonstrated a significant upregulation of circMYH9 in CRC tissues. This upregulation destabilizes p53 pre-mRNA by recruiting hnRNPA2B1 in the nucleus. hnRNPA2B1 binds to and stabilizes N6-methyladenosine in the 3′ untranslated region of p53 pre-mRNA. This, in turn, regulates serine/glycine metabolism and redox homeostasis, thereby promoting the proliferation of cancer cells. Moreover, *in vivo* transfection of circMYH9 mediated by adeno-associated virus serotype 9 (AAV9) can induce chemically driven carcinogenesis in mice by inhibiting p53 (Liu X. et al., 2021). In other study, Yang discovered that mucin 17 inhibits the progression of human gastric cancer by curbing the inflammatory response, a process mediated by the MYH9-p53-RhoA regulatory feedback loop (Yang et al., 2019).

##### 4.1.1.4 miRNA

A non-coding RNA called miRNA, which has 22–26 nucleotides, makes up 1% of the human genome’s total number of genes (Hill and Tran, 2021). One method by which eukaryotic cells control gene transcription is through binding to the untranslated 3′ UTR region of target genes, which in order to inhibit the target genes’ post-transcriptional activity, that in turn affects the level of gene expression and consequently intracellular homeostasis (Lopez-Camarillo et al., 2021). Numerous crucial biological processes, including cell differentiation, proliferation, apoptosis, and metabolism, can be controlled by miRNAs (Breulmann et al., 2023). The fourth deadliest cancer is CRC (Kong et al., 2023). According to the research, the cyclic RNA hsa_circ_0000395 (circ_0000395), which has been demonstrated to be elevated in CRC, can improve the production of MYH9 by chelating miR-432-5p, which in turn causes CRC to advance (Fan et al., 2023). In pancreatic ductal adenocarcinoma (PDAC) tissues, circSTX6 was found to be considerably elevated. Through the circSTX6/miR-449b-5p axis and the circSTX6/CUL2/HIF1A signaling pathway, circSTX6 controls MYH9 expression. Additionally, through interacting with CUL2, MYH9 transcription was sped up, boosting PDAC cell growth and metastasis (Meng et al., 2022; Gautam et al., 2023). The miR-138-5p/MYH9 axis boosted cisplatin resistance and decreased immune response in cancer cells in non-small cell lung cancer (NSCLC) (Xu et al., 2021; Wang S. et al., 2022). Circ_NEK6 gene was identified to control the miR-370-3p/MYH9 axis in differentiated thyroid carcinoma, increasing type I resistance (Chen et al., 2021). By increasing MYH9 expression *via* miR-186-5p in gastric cancer, circ-NRIP1 might speed up glycolysis and the disease’s progression (Liu Y. et al., 2020), and Fang et al. discovered that the miR-204-5p/MYH9 axis could similarly encourage glycolysis, cell migration, and invasion in GC cells (Fang et al., 2020). Additionally, GC growth was also aided by overexpression of the miR-9-5p/MYH9 axis (Liu T. et al., 2020).

#### 4.1.2 Association of MYH9 with tumor metastasis and prognosis

##### 4.1.2.1 MYH9 is involved in tumor proliferation, migration and infiltration

Recent studies increasingly demonstrate the involvement of MYH9 in cell growth, proliferation, tumor invasion, metastasis, and other significant roles in cancer (Babbin et al., 2009; Rai et al., 2017; Sun et al., 2022; Wu et al., 2023). NMIIA can form an apical actin network that influences cell division, migration and accelerates tumor progression (Figure 1). It is found in the prominent terminal and perinuclear regions of primary tumor cells (Shutova et al., 2014; Kuragano et al., 2018; Halder et al., 2019; Surcel and Robinson, 2019; Yamamoto et al., 2019).

In a study by Liu et al., MYH9 expression was significantly elevated in NSCLC (*p* < 0.001), and high expression was associated with significantly reduced patient survival (*p* = 0.023). Cellular experiments revealed that MYH9 knockdown significantly suppressed cell proliferation (*p* < 0.001) and enhanced apoptosis (*p* < 0.05) (Liu F. et al., 2023). In another investigation, Tang et al. reported that the YY1-FGL1-MYH9 axis regulated the proliferation of LUAD cells, consequently promoting tumor growth (Tang et al., 2022). Additionally, a separate study identified MICAL2, a tumor promoter, as a nucleoplasmic shuttle protein dependent on MYH9 and its C-terminal fragment. Experimental data showed that these two factors synergistically promoted the migration, invasion, deformation, and proliferation of LUAD cells (Zhou et al., 2020a). NM IIA can bind to the MYH10 protein, recruiting deubiquitin-specific protease 45, which deubiquitinates snail to prevent snail degradation. This process ultimately promotes proliferation, migration, invasion, metastasis, and cisplatin resistance in ovarian cancer (Liu L. et al., 2023). Human tubulin beta class IVa (TUBB4A), a member of the β-microtubulin family, is overexpressed in prostate cancer. MYH9 interacts with TUBB4A to safeguard the nucleus during cell migration, promoting the progression of prostate cancer *via* GSK 3β/β-catenin signaling (Gao et al., 2022). This pathway has also been linked to the postoperative recurrence of EC (Li Q. et al., 2023). Experimental evidence suggests that MYH9 acts as a novel androgen receptor co-repressor, playing a pivotal role in the progression of treatment-resistant prostate cancer (Liu C. et al., 2021). MYH9 is a potent promoter of tumor stem cells that can prompt hepatocellular carcinogenesis (Lin et al., 2020). Additionally, MYH9 can expedite the progression of HCC and EC through the PI3K/AKT/mTOR signaling pathway (Zhao R. et al., 2022; Kai et al., 2022). Increased phosphorylation of NM IIA and myosin-regulated light chains indicates enhanced actinomyosin contractility in various AML cell lines. Actinomyosin-mediated contractility is essential for processes such as cell division, adhesion, and migration (Chang et al., 2020). Autophagy-associated protein 9B (ATG9B) represents a crucial potential target gene for CRC metastasis. MYH9, which interacts significantly with ATG9B, facilitates colorectal cancer invasion through non-autophagic mechanisms (Zhong et al., 2021). A study has verified the capability of dNTPs to bind MYH9 with differing efficiencies. Additionally, cellular thermal shift analysis has demonstrated that dNTPs augment the thermal stability of MYH9. EdU labeling and flow cytometry-based cell cycle analysis have corroborated MYH9’s role in propelling cells into the S phase. This data implies a novel function for MYH9 involving dNTPs binding and its ability to facilitate DNA synthesis (Nangia-Makker et al., 2022).

MYH9 significantly influences tumor cell migration and infiltration. It regulates the motility and morphodynamics of tumor cells, actively engages in relevant signaling pathways, and interacts with other proteins to impact tumor proliferation, migration, and infiltration (Wang et al., 2011; Ivanov et al., 2022). Nonetheless, MYH9 assumes diverse roles across different types of tumors, each involving distinct mechanisms that warrant further investigation.

##### 4.1.2.2 High MYH9 expression is associated with tumor clinical stage, histological type

The expression levels of MYH9 were notably elevated in the pertinent tumor tissues and exhibited correlations with the clinical stage, histological type, disease progression, and an unfavorable prognosis of the tumor.

Li et al. observed that suppressing MYH9 reduced the stemness, EMT, angiogenesis, metastasis, and tumorigenicity of EC cells, implying a pro-tumorigenic role for MYH9 in EC, closely linked to tumor stage (Li Q. et al., 2023). A computational analysis aimed at predicting survival in colon cancer integrated data on copy number variations and gene expression, identifying pathogenic driver genes associated with patient prognosis. Within this analysis, a survival prediction model that incorporated the expression of three candidate genes, including MYH9, demonstrated superior predictive performance. Further functional analyses confirmed that the knockdown of MYH9 decreased the primary activity of colon cancer cells. Notably, validation using an independent cohort of colon cancer patients established that co-expression of MYH9 and other genes correlated with poorer clinical outcomes in terms of overall and disease-free survival (*p* < 0.001) (Lee C. J. et al., 2023). Collectively, these findings highlight a substantial association between MYH9, colon cancer tumor stage, and an unfavorable prognosis. Another study revealed that MYH9 regulates the content of lipid droplets (LDs) by binding to ARP2/3. The breakdown of LDs releases energy and supports cancer cell proliferation and migration. The number of LDs and the amount of triglycerides (TGs) increased following MYH9 intervention. Notably, the overexpression of ARP2/3 and MYH9 significantly elevated the expression of genes related to fatty acids and neutral lipid synthesis (*p* < 0.05). These changes were strongly linked to a poor prognosis in cervical squamous cell carcinoma (CSCC) (Zhao P. et al., 2022). This study provided insights into how cytoskeletal filaments affect LD metabolism in cancer cells. MYH9 also plays a role in glioma. A recent study observed increased MYH9 expression in gliomas, and this elevated expression was associated with WHO grading. Elevated MYH9 expression can drive the acquisition of a malignant phenotype in glioma cells and contribute to their resistance to chemotherapy. Furthermore, MYH9 interacts with GSK-3β, leading to the inhibition of GSK-3β protein expression through ubiquitination. Subsequently, the reduction of GSK-3β promotes the nuclear translocation of β-linker proteins, thereby enhancing glioma cell growth, invasion, migration, and resistance to temozolomide (Chen et al., 2023). The level of MYH9 expression significantly correlates with patient survival and should be considered as an independent prognostic indicator (Que et al., 2021). Katono and colleagues discovered a significant correlation between MYH9 expression and several factors: adenocarcinoma histology (*p* = 0.014), poor differentiation (*p* = 0.033), intratumoral vascular invasion (*p* = 0.013), lymphatic invasion (*p* = 0.045), and a poor prognosis (*p* = 0.032) (Katono et al., 2015).

Overexpression of MYH9 has a significant impact on tumor clinical staging through multiple mechanisms, resulting in a poorer prognosis. Elucidating MYH9’s mechanism of action can enhance the clinic’s ability to precisely stage tumors and evaluate prognosis across different cancer types, thereby improving the development of more effective therapeutic strategies for patients.

#### 4.1.3 Relationship between MYH9-RD and cancer

MYH9-RD typically denotes autosomal dominant disorders resulting from MYH9 mutations, with the exception of tumors (Smith et al., 2019; Bury et al., 2020; An et al., 2022). To date, a few case reports have suggested a potential link between MYH9-RD and certain tumors.

In a 19-year-old female harboring a germline MYH9 variant, a right tongue ulcer was detected, and a biopsy confirmed the presence of squamous cell carcinoma. At the age of 12, she had received a prior diagnosis of EPS, a form of MYH9-RD. This study postulates that MYH9-RD may manifest early as a progressively localized malignant oral cavity tumor (Yabe et al., 2022). Rheingold documented another case in which a child with a confirmed diagnosis of autosomal dominant megathrombocytopenia (FTNS) went on to develop AML (Rheingold, 2007). In a distinct AML cell line, NM IIA and myosin-regulated light chain phosphorylation levels were elevated (Chang et al., 2020). This led to speculation about a potential connection between these two conditions.

Currently, there is limited documentation on the connection between MYH9-RD and tumors, and isolated case reports do not provide sufficient evidence for a definitive correlation. However, they do hint at the need for clinical professionals to focus on this aspect and validate these assumptions by amassing a substantial number of clinical cases. Such an effort will be of immense importance for future patient prevention, long-term treatment, and prognosis.

### 4.2 MYH9 can act as a tumor suppressor gene

MYH9 may act as a tumor suppressor gene in specific cases. Alanazi et al. observed that inhibiting NM IIA promotes tumorigenesis in HER2+ breast cancers (Alanazi et al., 2023). Singh et al. demonstrated that reducing MYH9 expression in melanoma cells enhances *in vitro* migration and invasion. Moreover, MYH9 suppression accelerates tumor growth and metastasis in mouse models of melanoma. Oncogene analysis indicates MYH9’s regulation of EMT and the ERK signaling pathways. Additionally, MYH9 influences the tumor microenvironment (TME) by modulating leukocyte and macrophage infiltration in tumors, which suggests an unexpected role as a melanoma tumor suppressor (Singh et al., 2020). In the case of head and neck squamous cell carcinoma (HNSCC), a study found that low MYH9 expression correlates with decreased survival among HNSCC patients with low-risk mutp53. Furthermore, inhibiting NM IIA leads to increased invasion of cells containing wild-type p53 (wtp53), accompanied by reduced expression of p53 target genes. These findings imply that NM IIA acts as a tumor suppressor in HNSCC (Coaxum et al., 2017). Furthermore, a direct *in vivo* RNAi screen demonstrated that NM IIA acts as a tumor suppressor in squamous cell carcinoma (MYH9 regulates p53 stability and, 2014; Schramek et al., 2014). Ezrin-radixin-moesin-binding phosphor protein 50 (EBP 50) is a scaffolding protein required for epithelial polarity (Claperon et al., 2012; Du G. et al., 2016; Oh et al., 2017), and it was found that the interaction of membrane ebp 50 (Me-EBP50) and MYH9 is a favorable prognostic factor in ovarian clear cell carcinoma (Nakagawa et al., 2023).

Elucidating the suppressive functions of MYH9 and NM IIA in specific tumors can facilitate their clinical exploitation for more informed treatment strategies.

## 5 Tumor therapy for MYH9

MYH9 has been observed to be overexpressed in various tumors and plays a role in tumor development. Recent research has revealed that NM II, encoded by MYH9, serves as a crucial cytoskeletal protein that generates contractile forces essential for cell migration and subcellular component movement. This discovery positions NM II as a highly promising target for cancer therapy (Table 2).

**TABLE 2:** Therapies targeted MYH9.

Therapies	Tumors	Mechanism	References
Cinobufotalin	Lung adenocarcinoma	Upregulate the expression of ENKUR through the inhibition of PI3K/AKT/c-Jun-mediated transcriptional repression	Liu et al. (2022)
	Nasopharyngeal carcinoma		Hou et al. (2022b)
	Gastric cancer		Liu et al. (2020a)
DT-13	Gastric cancer	Combine with topotican promoted the degradation of epidermal growth factor receptor	Yu et al. (2019)
	Lung cancer	Inhibit human lung cancer metastasis under hypoxic condition	Wei et al. (2016)
	Breast cancer	Inhibit migration by regulating stromal cells in the TME	Gao et al. (2020b)
Immunotherapy	Lung adenocarcinoma	YY1-FGL1-MYH9 axis	Tang et al. (2022)
	Colorectal cancer	MAP7D2 interacting with MYH9, MAP7D2 knockdown increased the infiltration of CD8 CTLs, thereby inhibiting tumor progression	Wu et al. (2023)
Amidated fullerenes		Resulting in altered MYH9 localization, and also inhibiting metastasis-associated EMT	Huo et al. (2022), Li et al. (2023a), Zhou et al. (2020b)
J13		Weakening MYH9-actin interactions and deactivating the molecular motors to promotes the mitochondrial division process, leading to an imbalance in its dynamics and significantly inhibiting cancer cell survival, proliferation and migration	Qian et al. (2021)
ITE	Glioma	Agonizing endogenous aromatic hydrocarbon receptors and blocks multiple modes of cell migration	Zhao et al. (2020)
Astrocystin	Gastric cancer	Targeting cytosolic MYH9-induced CTNNB1 transcription to promote anti-apoptosis as well as metastasis	Ye et al. (2020)
Apatinib	Glioma	Target platelet-responsive protein 1 (THBS1), thereby inhibit glioma cell malignancy through its interaction with MYH9	Yao et al. (2021)

### 5.1 MYH9 promotes tumor drug resistance

Studies have revealed a connection between MYH9 and tumor drug resistance. Elevated MYH9 levels can modulate the NOTCH pathway, promoting resistance to Levatinib in HCC (Yang et al., 2023). It has been shown that increasing extracellular matrix stiffness not only alters the malignant characteristics of HCC cells, but also attenuates the efficacy of Metformin treatment (Gao X. et al., 2020). Interestingly, Gao et al. found that 354 differential membrane proteins, including MYH9, may be associated with stromal stiffness-mediated Metformin resistance (Gao et al., 2023). LincROR plays a crucial role in regulating tumorigenesis and metastasis (Lee Y. H. et al., 2023; Li S. Y. et al., 2023; Jiang et al., 2023; Mazur et al., 2023). Jiang et al. discovered that lincROR interacts with and stabilizes MYH9, enhancing the β-conjugated protein/HIF1α pathway, creating a positive feedback loop of lincROR/MYH9/HIF1α, and thus, promoting Docetaxel resistance in prostate cancer (Jiang et al., 2023). Furthermore, NM IIA can shield colon cancer cells from 5-FU-induced apoptosis and inhibition of proliferation through the AMPK/mTOR pathway (Wang et al., 2021). A case report on LUAD identified a novel MYH9-RETA fusion and T790M deletion in plasma circulating tumor DNA (ctDNA) following Osimotinib treatment, leading to rapid progression after 5 months and suggesting a potential resistance mechanism (Sun et al., 2020). Piskareva et al. found that elevated MYH9 levels induce EMT in neuroblastoma cells (Piskareva et al., 2015), a crucial feature in the development of cisplatin resistance in neuroblastoma (Xu et al., 2020; Belhajova et al., 2022; Li H. et al., 2022).

However, several studies have demonstrated the potential of the small molecule compound CB to reverse chemotherapeutic drug resistance associated with MYH9. It achieves this by inhibiting MYH9 transcription through the suppression of PI3K/AKT signaling, resulting in the downregulation of c-Jun, a negative transcription factor for ENKUR, leading to enhanced ENKUR expression. The reduced MYH9 levels diminish the recruitment of the deubiquitinating enzyme USP7, which in turn increases c-Myc ubiquitination and degradation, decreases c-Myc nuclear translocation, and deactivates the EMT signaling, thereby mitigating cisplatin resistance in LUAD (Liu et al., 2022). Additionally, it disrupts the interaction with its binding partner MYH9, effectively inducing FOXO1-mediated cisplatin sensitivity in nasopharyngeal carcinoma (Li et al., 2019). Liu et al. also observed that CB stimulates MAP2K4, subsequently inhibiting the MYH9/GSK3β/β-catenin pathway and downstream tumor stem cell and EMT signaling, resulting in a significant reversal of EBV-Mir-BarT2-induced cisplatin resistance in nasopharyngeal carcinoma (Liu Y. et al., 2019).

While current studies demonstrate that CB can reverse tumor drug resistance associated with MYH9, its applicability is limited to a few cancer types, and further investigation is required to determine its effectiveness in other cancers. Additionally, the exploration of other drugs with superior reversal properties necessitates more in-depth research. However, these strategies primarily address drug resistance once it has already developed. To guide future research, the central focus should be on elucidating the mechanisms of drug resistance, resolving its underlying causes, and seeking more effective drugs for cancer treatment.

### 5.2 Small molecule drugs targeting MYH9

#### 5.2.1 ENKUR and CB

ENKUR has been identified as a tumor suppressor encoding Enkurin protein which plays a crucial role in intracellular signaling by interacting with transient receptor potential cation channel (TRPC) (Ma et al., 2019a; Ma et al., 2019b). Additionally, chemically synthesized CB has demonstrated significant anticancer effects on specific tumors (Li et al., 2019; Li et al., 2021; Li W. et al., 2022; Wang J. et al., 2022), and there might be interactions between these two factors. Hou et al. conducted a study revealing that CB, as a safe and effective anticancer compound, can enhance ENKUR expression by inhibiting PI3K/AKT/c-Jun-mediated transcriptional repression. ENKUR or its Enkurin structural domain binds to MYH9, reducing its expression by binding to β-catenin and inhibiting its nuclear translocation, consequently lowering c-Jun levels. This, in turn, inhibits the β-catenin/c-Jun/MYH9 signaling pathway. The decreased MYH9 expression hinders the recruitment of the deubiquitylating enzyme USP7, promoting c-Myc degradation and, subsequently, inhibiting cell cycle progression and EMT signaling (Hou et al., 2022a). Moreover, Liu et al. found that CB can also inhibit MYH9-mediated c-Myc deubiquitination by inducing ENKUR expression for therapeutic purposes in LUAD (Liu et al., 2022). In nasopharyngeal carcinoma, CB-induced ENKUR similarly inhibited β-catenin/c-Jun/MYH9 signaling, reducing UBE3A-mediated p53 ubiquitination and degradation (Hou et al., 2022b). Furthermore, ENKUR’s binding to MYH9 reduces its protein expression by recruiting the E3 ubiquitin ligase FBXW7 to form a ubiquitination degradation complex. The downregulated MYH9 protein impairs the recruitment of the deubiquitinase USP2, promoting the degradation of β-conjugated proteins and ultimately inhibiting EMT signaling, cell migration, invasion, and metastasis, indicating its potential as a therapeutic target in gastric cancer (Liu J. et al., 2020). However, whether CB can upregulate ENKUR expression for therapeutic purposes in gastric cancer has not been reported.

#### 5.2.2 DT-13

DT-13, known as saponin monomer 13, is a bioactive compound derived from maitake (Du H. et al., 2016; Khan et al., 2018). It has been reported to effectively inhibit the metastasis of various types of cancers (Du H. et al., 2016; Wang et al., 2018; Wei et al., 2019). When combined with topotecan (TPT), DT-13 promotes the degradation of the epidermal growth factor receptor (EGFR) by inducing EGFR endocytosis through NM IIA. This process further inhibits the activity of hexokinase II (HK II). Consequently, DT-13 enhances the suppression of aerobic glycolysis in BGC-823 cells, ultimately achieving a more effective inhibitory effect on tumors (Yu et al., 2019). Wei et al. also discovered that, under hypoxic conditions, DT-13 hinders the metastasis of human lung cancer by regulating NM IIA activity (Wei et al., 2016). In the TME, cancer cell migration is promoted by the regulation of NM IIA expression. DT-13 combats cancer cell migration in TME models by inhibiting the c-raf/ERK1/2 signaling pathway. This inhibition, in turn, reduces NM IIA expression, effectively blocking cancer cell migration (Du H. et al., 2016). Furthermore, DT-13 inhibits breast cancer cell migration by influencing the MYH9 gene in stromal cells within the TME (Gao Y. et al., 2020).

### 5.3 MYH9 and immunotherapy

Immunotherapy is an emerging cancer treatment method that involves modulating the patient’s own immune system to enable it to more effectively identify and eliminate abnormal cells in the body. Immunotherapies targeting MYH9 have also become relevant in cancer treatment. Lung cancer holds the unenviable title of being the most prevalent tumor worldwide, with the highest mortality rate and the second-highest incidence rate (Siegel et al., 2023). Among the various approaches for treating LUAD, immunotherapy stands out as one of the most crucial (Song P. et al., 2022; Hao et al., 2022). To explore the potential of fibrinogen-like protein 1 (FGL1) as a therapeutic option for LUAD, Tang et al. conducted a study involving 200 LUAD patients. Their findings revealed that FGL1 can modulate the secretion of the vital immune-related cytokine YY1-FGL1-MYH9 axis, thereby influencing its impact on LUAD (Tang et al., 2022). In CRC patients with microsatellite-stable (MSS) tumors, the limited presence of CD8 cytotoxic T lymphocytes (CTLs) significantly constrains treatment options. In both *in vitro* and *in vivo* experiments, knocking down MAP7D2 resulted in a notable increase in CD8 CTL infiltration, leading to the inhibition of tumor progression. Subsequent investigations unveiled that the interaction between MAP7D2 and MYH9 shields MAP7D2 from ubiquitin-mediated degradation and subsequently reduces HMGB1 secretion. This, in turn, inhibits CD8 CTL infiltration in MSS CRC. These findings suggest that targeting MAP7D2 in MSS CRC could present a novel avenue for anti-tumor immunotherapy (Wu et al., 2023). Another study revealed that perforin interacts with non-muscle MYH9 to exert force on the lesser F-actin in tumor regenerating cells (TRCs). This interaction results in the stiffening of TRCs and enables perforin to penetrate the cell membrane, facilitating CTL-mediated killing of TRCs and promoting tumor immunotherapy (Liu Y. et al., 2021).

### 5.4 Other anti-tumor methods

Amidated fullerenes exhibit significant antitumor effects. The synthesized amphiphilic derivative of fullerene, TAPC-4, possesses a well-defined molecular structure and amphiphilic properties, with a terminal amino group that enhances its ability to target MYH9 (Shin et al., 2011). This targeting may lead to altered MYH9 localization (Huo et al., 2022) and inhibition of metastasis-associated EMT (Zhou et al., 2020b). Apatinib targets platelet-responsive protein 1 (THBS1) in glioma cells, thereby inhibiting glioma cell malignancy through its interaction with MYH9 (Yao et al., 2021). Qian et al. discovered that the naturally sourced small molecule J13 can directly target the MYH9-actin molecular motors. By weakening MYH9-actin interactions and deactivating these molecular motors, it promotes the mitochondrial division process, resulting in an imbalance in mitochondrial dynamics and a significant inhibition of cancer cell survival, proliferation, and migration (Qian et al., 2021). Another study revealed that the small molecule, methyl 2-(1H-indole-3-carbonyl)-thiazole-4-carboxylate (ITE), activates endogenous aromatic hydrocarbon receptors (AHR) and hinders various modes of glioma cell migration (Zhao et al., 2020). Astrocystin can target cytosolic MYH9-induced CTNNB1 transcription, promoting anti-apoptosis and metastasis of gastric cancer cells. This offers a novel therapeutic approach for peritoneal metastasis of gastric cancer (Ye et al., 2020). Additionally, miRNAs play a role in targeting MYH9-related signaling pathways (Ye et al., 2017; Ye et al., 2018; Hart et al., 2019; Liu L. et al., 2020; Chen et al., 2021).

## 6 Conclusion

MYH9 gene encodes NM IIA, which was previously considered a constituent of the cytoskeleton, providing cellular support and facilitating intracellular transport. However, in recent years, an increasing body of evidence has revealed that NM IIA participates in numerous pathophysiological processes and even plays a pivotal role in the onset and development of tumors. This paper offers a summary, but several unresolved issues and divergent perspectives endure. Further investigation is required to elucidate the mechanism by which MYH9 affects tumor proliferation, infiltration, and migration. Equally important is the exploration of its role in driving drug resistance in tumors. The current understanding of the connection between MYH9-RD and tumors remains unclear, and the limited number of case reports fails to offer conclusive evidence. This situation also requires clinical doctors' attention in their practical work, accumulating relevant cases, so as to accurately determine the correlation between MYH9-RD and tumors. Nonetheless, unraveling this relationship holds immense significance for future disease prevention, long-term patient treatment, and prognosis. While the question of whether MYH9 functions as an oncogene or a tumor suppressor gene is contingent upon the specific tumor type, additional research is warranted. Given the unique role that MYH9 plays in tumors, it stands as a highly promising and effective target for cancer therapy. And the new therapies still needed to be explored. The gene can also be used in early diagnosis, clinical staging and prognosis of cancers. If more studies focus on these points, they will bring great benefits. This review summarizes recent research on MYH9’s role in tumors, with the hope that it can provide insights and references for future clinical studies.

## Figures and Tables

**Table:** 

**MYH9**	myosin heavy chain 9
**NM IIA**	non-myosin heavy chain IIA
**CB**	Cinobufotalin
**DT-13**	saponin monomer 13
**NMMHC IIA**	non-muscle myosin heavy chain IIA
**ROCK1**	Rho-kinase 1
**RLC**	Rho light chain
**NM II**	non-myosin II
**EMT**	epithelial-mesenchymal transition
**Pi**	Phosphoric acid
**MNA**	May-Hegglin anomaly
**EPS**	Epstein syndrome
**FTNS**	Fechtner syndrome
**SBS**	Sebastian syndrome
**HCC**	hepatocellular carcinoma
**AML**	acute myeloid leukemia
**EC**	esophageal cancer
**ccRCC**	clear cell renal cell carcinoma
**CRC**	colorectal cancer
**AAV9**	adeno-associated virus serotype 9
**GC**	gastric cancer
**PDAC**	pancreatic ductal adenocarcinoma
**NSCLC**	non-small cell lung cancer
**TUBB4A**	tubulin beta class IVa
**ATG9B**	autophagy-associated protein 9B
**LDs**	lipid droplets
**TGs**	triglycerides
**CSCC**	cervical squamous cell carcinoma
**HNSCC**	head and neck squamous cell carcinoma
**wtp 53**	wild-type p53
**EBP50**	ezrin-radixin-moesin-binding phosphor protein 50
**Me-EBP50**	membrane EBP 50
**ctDNA**	circulating tumor DNA
**HIF1α**	hypoxia-inducible factor 1-alpha
**TPT**	topotecan
**EGFR**	epidermal growth factor receptor
**HK II**	hexokinase II
**TME**	tumor microenvironment
**FGL 1**	fibrinogen-like protein 1
**MSS**	microsatellite-stable
**CTLs**	cytotoxic T lymphocytes
**LUAD**	lung adenocarcinoma
**TRCs**	tumor regenerating cells
**THBS 1**	apatinib targets platelet-responsive protein 1
**ITE**	methyl 2-(1H-indole-3-carbonyl)-thiazole-4-carboxylate
**AHR**	aromatic hydrocarbon receptors
**EC**	esophagus cancer
